# Response Ant Colony Optimization of End Milling Surface Roughness

**DOI:** 10.3390/s100302054

**Published:** 2010-03-15

**Authors:** K. Kadirgama, M. M. Noor, Ahmed N. Abd Alla

**Affiliations:** 1 Faculty of Mechanical Engineering, University Malaysia Pahang, Kuantan, 26300 UMP, Malaysia; E-Mail: muhamad@ump.edu.my; 2 Faculty of Electrical and Electronic Engineering, University Malaysia Pahang, Kuantan 26300 UMP, Malaysia; E-Mail: ahmed@ump.edu.my

**Keywords:** response surface method, ant colony, aluminium alloys, surface roughness

## Abstract

Metal cutting processes are important due to increased consumer demands for quality metal cutting related products (more precise tolerances and better product surface roughness) that has driven the metal cutting industry to continuously improve quality control of metal cutting processes. This paper presents optimum surface roughness by using milling mould aluminium alloys (AA6061-T6) with Response Ant Colony Optimization (RACO). The approach is based on Response Surface Method (RSM) and Ant Colony Optimization (ACO). The main objectives to find the optimized parameters and the most dominant variables (cutting speed, feedrate, axial depth and radial depth). The first order model indicates that the feedrate is the most significant factor affecting surface roughness.

## 1. Introduction

Roughness plays an important role in determining how a real object will interact with its environment. Rough surfaces usually wear more quickly and have higher friction coefficients than smooth surfaces. Roughness is performance of a mechanical component, since irregularities in the surface may form nucleation soften a good prediction for cracks or corrosion. Although roughness is usually undesirable, it is difficult and expensive to control in manufacturing. Decreasing the roughness of a surface will usually exponentially increase its manufacturing costs. This often results in a trade-off between the manufacturing cost of a component and its performance in an application.

Planning of experiments through design of experiments has been used quite successfully in process optimization by Chen and Chen [1], Fung and Kang [2], Tang *et al.* [3], Vijian and Arunachalam [4], Yang [5] as well as Zhang *et al.* [6], *etc*. Four controlling factors including the cutting speed, the feed rate, the depth of cut, and the cutting fluid mixture ratios with three levels for each factor were selected. The Grey relational analysis is then applied to examine how the turning operation factors influence the quality targets of roughness average, roughness maximum and roundness. An optimal parameter combination was then obtained. Additionally, ANOVA was also utilized to examine the most significant factors for the turning process when the roughness average, roughness maximum and roundness are simultaneously considered.

Aslan *et al.*, [7], using Design optimization of cutting parameters when turning hardened AISI 4140 steel (63 HRC) with Al_2_O_3_ + TiCN mixed ceramic tool used an orthogonal array and the analysis of variance (ANOVA) to optimization of cutting parameters. The flank wear (VB) and surface roughness (Ra) had investigated. Nalbant *et al.* [8] used a Taguchi method to find the optimal cutting parameters for surface roughness in turning operations of AISI 1030 steel bars using TiN coated tools. Three cutting parameters, namely, insert radius, feed rate, and depth of cut, are optimized with considerations of surface roughness, and so on. However, very few studies have been conducted to investigate roundness under different turning parameters. Additionally, proper application of cutting fluids as studied by Kalpakjian and Schmid, [9] and EI Baradie, [10], can increase productivity and reduce costs by allowing one to choose higher cutting speeds, higher feed rates and greater depths of cut. Effective application of cutting fluids can also increase tool life, decrease surface roughness, increase dimensional accuracy and decrease the amount of power consumed. Water-soluble (water-miscible) cutting fluids are primarily used for high speed machining operations because they have better cooling capabilities [10]. There fluids are also best for cooling machined parts to minimize thermal distortions. Water-soluble cutting fluids are mixed with water at different ratios depending on the machining operation. Therefore, the effect of water-soluble cutting fluids under different ratios was also considered in this study.

A recent investigation performed by Alauddin *et al.* [11] has revealed that when the cutting speed is increased, productivity can be maximised and, meanwhile, surface quality can be improved. According to Hasegawa *et al.* [12], surface finish can be characterised by various parameters such as average roughness (Ra), smoothening depth (Rp), root mean square (Rq) and maximum peak-to-valley height (Rt). The present study uses average roughness (Ra) for the characterisation of surface finish, since it is widely used in industry. By using factors such as cutting speed, feed rate and depth of cut, Hashmi and his coworkers [13,14] have developed surface roughness models and determined the cutting conditions for 190 BHN steel and Inconel 718. EI-Baradie [15] and Bandyopadhyay [16] have shown that by increasing the cutting speed, the productivity can be maximised and, at the same time, the surface quality can be improved. According to Gorlenko [17] and Thomas [18], surface finish can be characterised by various parameters. Numerous roughness height parameters such as average roughness (Ra), can be closely correlated. Mital and Mehta [19] have conducted a survey of the previously developed surface roughness prediction models and factors influencing the surface roughness. They have found that most of the surface roughness prediction models have been developed for steels.

## 2. Theoretical Background

### 2.1. Response Surface Method

This is a method for obtaining an approximate function using results of several numerical calculations to increase calculation efficiency and thereby implement design optimization. In the response surface method, design parameters are changed to formulate an approximate equation by the design of experiments method. An approximate sensitivity calculation of a multicrestedness problem can be performed using a convex continuous function and applied to optimization. The Box-Behnken Design is normally used when performing non-sequential experiments. That is, performing the experiment only once. These designs allow efficient estimation of the first and second–order coefficients. Because Box-Behnken designs have fewer design points, they are less expensive to run than central composite designs with the same number of factors. Box-Behnken designs do not have axial points, thus we can be sure that all design points fall within the safe operating zone. Box-Behnken designs also ensure that all factors are never set at their high levels simultaneously [20–22]. The proposed linear model correlating the responses and independent variables can be represented by the following expression:

(1) y=mCuttingspeed+nFeedrate+pAxialdepth+C

where *y* is the response, *C, m, n* and *p* are the constants Equation (1) can be written in the Equation (2):

(2) $$y=\beta_{0}x_{0}+\beta_{1}x_{1}+\beta_{2}x_{2}+\beta_{3}x_{3}$$

where *y* is the response, *x_0_* = 1(dummy variable), *x_1_*= cutting speed, *x_2_* = feedrate, and *x_3_* = axial depth. *β_0_* = C and *β_1_*, *β_2_*, and *β_3_*, are the model parameters. The second-order model can be expressed as shown in Equation (3):

(3) $$y^{\prime \prime }=\beta_{0}x_{o}+\beta_{1}x_{1}+\beta_{2}x_{2}+\beta_{3}x_{3}+\beta_{11}x^{2}1+\beta_{22}x^{2}2+\beta_{33}x^{2}3+\beta_{11}x_{1}x_{2}+\beta_{12}x_{1}x_{3}+\beta_{14}x_{2}x_{3}$$

### 2.2. Ant Colony Optimisation

Ant colony optimization algorithms are part of swarm intelligence, that is, the research field that studies algorithms inspired by the observation of the behaviour of swarms. Swarm intelligence algorithms are made up of simple individuals that cooperate through self-organization, that is, without any form of central control over the swarm members. A detailed overview of the self organization principles exploited by these algorithms, as well as examples from biology, can be found in [23].

One of the first researchers to investigate the social behaviour of insects was the French entomologist Pierre-Paul Grassé. In the 1940s and 1950s, he was observing the behaviour of termites in particular, the *Bellicositermes natalensis* and *Cubitermes* species. He discovered [24] that these insects are capable of reacting to what he called “significant stimuli,” signals that activate a genetically encoded reaction. He observed [24] that the effects of these reactions can act as new significant stimuli for both the insect that produced them and for the other insects in the colony.

Goss *et al.* [25] developed a model to explain the behaviour observed in the binary bridge experiment. Assuming that after t time units since the start of the experiment, m_1_ ants had used the first bridge and m_2_ the second one, the probability p_1_ for the (m + 1)^th^ ant to choose the first bridge can be given by Equation (4):

(4) $$P_{1(m+1)}=\frac{{\left(m_{1}+k\right)}^{h}}{{\left(m_{1}+k\right)}^{h}+{\left(m_{2}+k\right)}^{h}}$$

where parameters k and h are needed to fit the model to the experimental data. The probability that the same (m + 1)^th^ ant chooses the second bridge is *P*_2(m+1)_ = 1 − *P_1_*_(m+1)_. Monte Carlo simulations, run to test whether the model corresponds to the real data [10], showed very good fit for k ≈ 20 and h ≈ 2. This basic model, which explains the behaviour of real ants, may be used as an inspiration to design artificial ants that solve optimization problems defined in a similar way.

Ant colony optimization has been formalized into a combinatorial optimization metaheuristic by Dorigo *et al.* [26,27] and has since been used to tackle many combinatorial optimization problems (COPS). Given a COP, the first step for the application of ACO to its solution consists in defining an adequate model. This is then used to defined the central component of ACO: the pheromone model. The model of a COP may be defined as follows:

A model *P* = *(S, Ω, f)* of a COP consists of:

- a search space S defined over a finite set of discrete decision variables and a set *Ω* of constraints among the variables;
- an objective function f: $S\to {𝒭}_{0}^{+}$ to be minimized

Ant System was the first ACO algorithm to be proposed in the literature [28–30]. Its main characteristic is that the pheromone values are updated by all the ants that have completed the tour. The pheromone update for τ_ij_, that is, for edge joining cities i and j, is performed as follows [Equation (5)]:

(5) $$T_{\text{ij}}\leftarrow (1-\rho ).T_{\text{ij}}+\sum_{k=1}^{m}{\Delta T}_{\text{ij}}^{k}$$

where *ρ* is the evaporation rate, *m* is the number of ants, and ${\Delta T}_{\text{ij}}^{k}$ is the quantity of pheromone per unit length laid on edge (*i, j*) by the *k*th ant [28] as shown in Equation (6):

(6) $${\Delta T}_{\text{ij}}^{k}=\{\begin{array}{ll} \frac{Q}{L_{k}}, & \mathrm{if ant k used edge}\;(i,j)\;\mathrm{in its tour}\\ 0, & \mathrm{otherwise} \end{array}$$

where *Q* is a constant and *L_k_* is the tour length of the kth ant.

## 3. Experimental Setup

**Figure 1.**
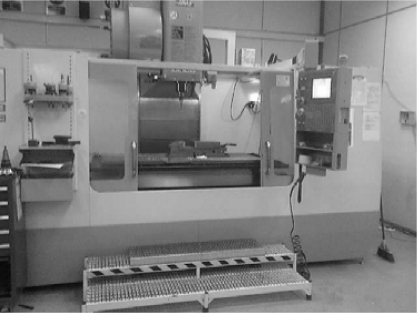
Haans CNC milling with 6-axes.

The 27 experiments were carried out on a 6-axes Haans machining centre as shown in Figure 1. A water soluble coolant was used in these experiments. Each experiment was stopped after 90 mm cutting length. For the surface roughness measurement surface roughness tester was used. Each experiment was repeated three times using a new cutting edge every time to obtain accurate readings of the surface roughness. The physical and mechanical properties of the workpiece are shown in Table 1 and Table 2. After the preliminary investigation, the suitable levels of the factors are used in the statistical software to deduce the design parameters for the aluminium alloy (AA6061-T6) as shown in Table 3. The lower and higher speed values selected are 100 m/s and 180 m/s, respectively. For the feed, the lower value is 0.1 mm/rev and the higher value is 0.2 mm/rev. For the axial depth, the higher value is 0.2 mm and the lower value is 0.1 mm and for the radial depth the higher value is 5 mm and lower value is 2 mm.

## 4. Results and Discussion

After conducting the first pass (one pass is equal to 90 mm length) of the 27 cutting experiments, the surface roughness readings are used to find the parameters appearing in the postulated first order model (Equation 1). In order to calculate these parameters, the least squares method is used with the aid of Minitab. The first-order linear equation used to predict the surface roughness is expressed by Equation 7:

(7) $$R_{a}=0.5764+0.0049C_{\mathrm{speed}}+3.5850f+1.5383a_{\mathrm{depth}}+0.016r_{\mathrm{depth}}$$

where the *C_speed_*, *f*, *a_depth_* and *r_depth_* are the cutting speed, feed rate, axial depth and radial depth respectively.

**Figure 2.**
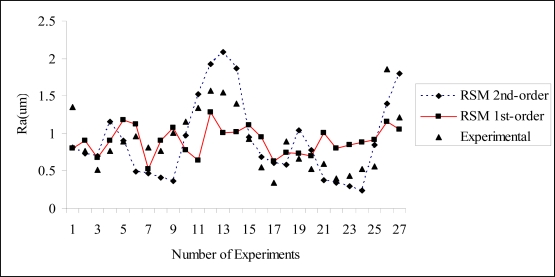
Comparison between the experimental and predicted results.

**Figure 3.**
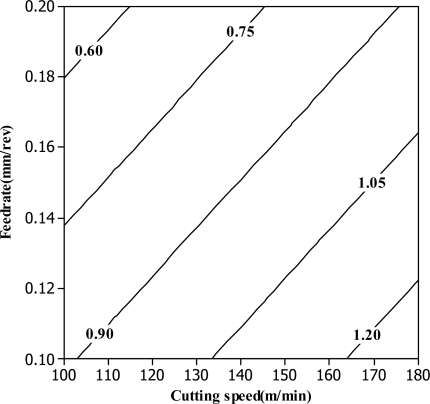
Feed rate *versus* cutting speed contour plotted for first-order model.

Generally, reduction of cutting speed, axial depth of cut caused a larger surface roughness. On the other hand, the increase in feed rate and radial depth caused a slight reduction of surface roughness. The feed rate is the most dominant factor on the surface roughness, followed by the axial depth, cutting speed and radial depth, respectively. Hence, a better surface roughness is obtained with the combination of low cutting speed and axial depth, high feed rate and radial depth. Similar to the first-order model, by examining the coefficients of the second-order terms, the feedrate (*f*) has the most dominant effect on the surface roughness. After examining the experimental data, it can be seen that the contribution of cutting speed (*C_speed_*) is the least significant. As seen from Figure 2, the predicted surface roughness using the second order RSM model is closely matched with the experimental results. It exhibits better agreement compared to those from the first-order RSM model. A contour plot of feed rate *versus* cutting speed for the first-order model is shown in Figure 3. It is clear that the relationship between the surface roughness and design variables can be obtained. The analysis of variance (ANOVA) for first order is tabulated in Table 4. It indicates that the model is adequate as the *P*-value of the lack-of-fit is not significant (>0.05).

## 5. Test Validation

**Figure 4.**
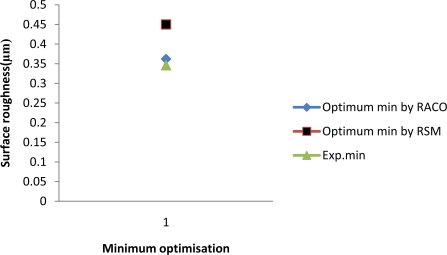
Comparison of minimum optimised surface roughness with experimental and RSM.

The optimised surface roughness model is tested with experimental results. The predicted minimum surface roughness using optimised surface roughness model by RACO are compared with the measured surface roughness and these results are reported in Figure 4. The validation experiment is performed in the same machining environment as the training experiment. The errors of surface roughness obtained by optimised min surface roughness model are 4.65%. The optimum cutting parameters for minimum surface roughness are cutting speed 100 m/min; feed rate 0.2 mm/rev, axial depth 0.1 mm and radial depth 5 mm. On the other hand, the optimisation by RSM is 0.45 μm [31].

## 6. Conclusions

This research illustrates the machining of aluminium alloy (AA6061-T6) with end-milling methods and predicting their subsequent surface roughness. There is becoming a need for investigating the machining of various types of aluminium and their surface roughness, which in turn can be useful in developing more cost effective personalised products. The authors have shown the use of RACO to formulate an optimised minimum surface roughness prediction model for end machining of AA6061-T6. This prediction model is tested on the validation experimental and the error analysis of the prediction result with the measured results is estimated at 4.65% for minimum surface roughness which is small and shows the efficacy of the prediction model. Finally, the simulation results show that ACO combine with RSM can be very successively used for reduction of the effort and time required. This means that it can solve many problems that have mathematical and time constraints.

## Figures and Tables

**Table 1.** Physical properties for workpiece.

**Component**	**Al**	**Cr**	**Cu**	**Fe**	**Mg**	**Mn**	**Si**	**Ti**	**Zn**
**Wt %**	95.8–98.6	0.04–0.35	0.15–0.4	Max 0.7	0.8–1.2	Max 0.15	0.4–0.8	Max 0.15	Max 0.25

**Table 2.** Design Parameters.

**Cutting speed (m/min)**	**Feedrate (mm/rev)**	**Axial depth (mm)**	**Radial depth (mm)**
140	0.15	0.10	5.0
140	0.15	0.15	3.5
100	0.15	0.15	5.0
140	0.15	0.15	3.5
180	0.15	0.20	3.5
180	0.15	0.15	2.0
100	0.20	0.15	3.5
140	0.15	0.15	3.5
180	0.15	0.15	5.0
100	0.15	0.20	3.5
140	0.20	0.10	3.5
180	0.10	0.15	3.5
140	0.15	0.20	2.0
180	0.15	0.10	3.5
140	0.10	0.15	2.0
140	0.15	0.20	5.0
100	0.15	0.10	3.5
140	0.20	0.15	2.0
100	0.15	0.15	2.0
140	0.20	0.15	5.0
140	0.10	0.10	3.5
140	0.20	0.20	3.5
140	0.15	0.10	2.0
100	0.10	0.15	3.5
180	0.20	0.15	3.5
140	0.10	0.20	3.5
140	0.10	0.15	5.0

**Table 3.** Mechanical properties of the workpiece.

Hardness, Brinell	95
Hardness, Knoop	120
Hardness, Rockwell A	40
Hardness, Rockwell B	60
Hardness, Vickers	107
Ultimate Tensile Strength	310 MPa
Tensile Yield Strength	276 MPa
Elongation at Break	12 %
Elongation at Break	17 %
Modulus of Elasticity	68.9 GPa
Density	2.7 g/cc

**Table 4.** Analysis of variance for first-order equation.

**Source**	**DOF**	**Seq. SS**	**Adj. SS**	**Adj. MS**	***F***	***P***
Regression	4	0.9309	0.9309	0.2327	0.78	0.552
Linear	4	0.9309	0.9309	0.2327	0.78	0.552
Residual Error	22	6.5937	6.5937	0.2997		
Lack-of-Fit	20	6.3151	6.3151	0.3158	2.27	0.351
Pure Error	2	0.2786	0.2786	0.1393		
Total	26	7.5246				
